# 1038. Assessment of risk factors for coronavirus disease 2019 in healthcare workers: a nested case-control study, Bishkek, Kyrgyzstan, June 2020-May 2021

**DOI:** 10.1093/ofid/ofac492.879

**Published:** 2022-12-15

**Authors:** Venera Alymkulova, Dilyara Nabirova, Nasyat Kemelbek kyzy, Dinara Otorbaeva, Chynar Zhumalieva, Nazgul Abamuslimova

**Affiliations:** Central Asia Field Epidemiology Training Program, Bishkek, Kyrgyzstan, Bishkek, Bishkek, Kyrgyzstan; US Centers for Disease Control and Prevention, Regional Office of Central Asia, Almaty, Kazakhstan, Almaty, Almaty, Kazakhstan; Central Asia Field Epidemiology Training Program, Bishkek, Kyrgyzstan, Bishkek, Bishkek, Kyrgyzstan; Department of Disease Prevention and State Sanitary and Epidemiological Supervision, Bishkek, Kyrgyzstan, Bishkek, Bishkek, Kyrgyzstan; Republican Scientific and Practical Center for Infectious Control of Scientific and Production Association "Preventive Medicine", Bishkek, Kyrgyzstan, Bishkek, Bishkek, Kyrgyzstan; Department of Disease Prevention and State Sanitary and Epidemiological Supervision, Bishkek, Kyrgyzstan, Bishkek, Bishkek, Kyrgyzstan

## Abstract

**Background:**

Despite infection control and mitigation measures against coronavirus disease (COVID-19) in Kyrgyzstan hospitals, 3173 healthcare workers (HCW) had been diagnosed with COVID-19 by September 2020 (22 COVID-19 cases/100 HCW). We aimed to identify risks for COVID-19 among HCW exposed to COVID-19 patients in Bishkek.

**Methods:**

We conducted a case-control study in six hospitals with high COVID-19 incidence using incidence-density sampling. Cases were HCW with positive SARS-CoV-2 PCR and negative SARS-CoV-2 IgG results June 2020-May 2021 exposed to COVID-19 patients < 14 days prior to PCR positive test. Controls were randomly selected HCW working with COVID-19 patients at the same time with negative SARS-CoV-2 PCR and IgG results. HCW with COVID-19 contacts outside of the work setting were excluded. We used logistic regression to identify factors associated with COVID-19.

**Results:**

We included 132 cases and 406 controls; 479 (89%) were women, and 256 (48%) were ages < 40 years. HCW wore different types of masks; among these medical respirators (FFP3) were used by 49% of cases vs 28% of controls. Comorbidities were reported in 34% of cases vs 14% of controls; 90% of cases vs 91% of controls stayed in shift-dormitories. Odds of COVID-19 were greater for HCW who used FFP3 vs other types of respirators including N95 (adjusted odds ratio=2.9, 95% confidence interval [CI]: 1.1–3.9), shared a shiftwork dormitory with another HCW diagnosed with COVID-19 vs not (2.9, CI: 1.5–5.5), and had a comorbidity vs not (2.3, CI:1.4–5.0).

**Figure:**
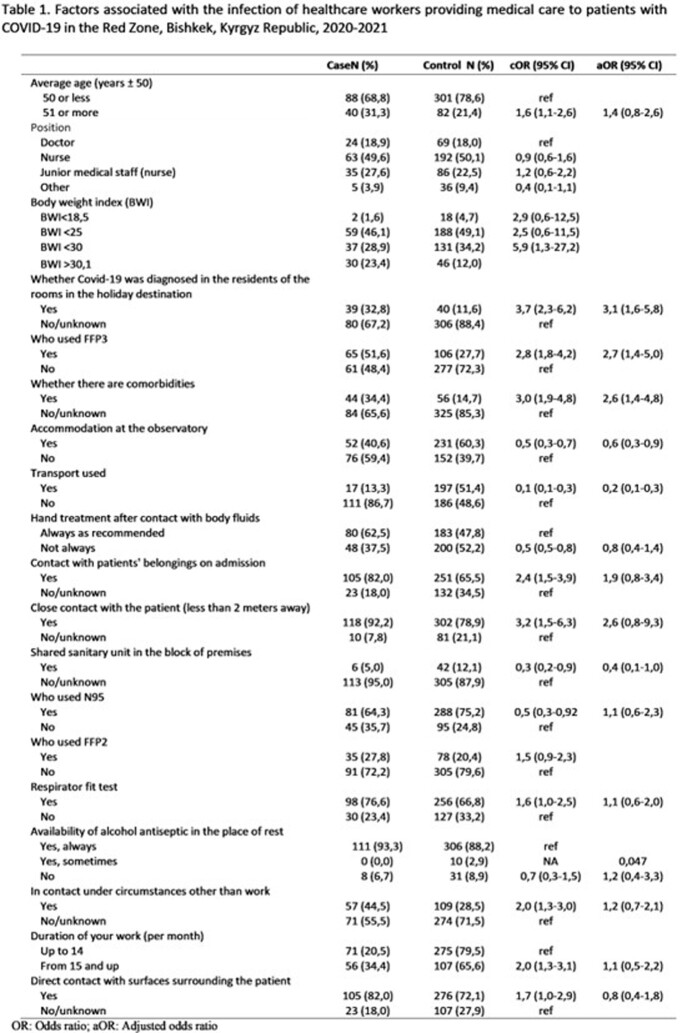

**Conclusion:**

HCW can become infected from exposures in a variety of settings. Our findings showed an association of COVID-19 with shared dormitory, comorbidity and FFP3 use. Results are not causal, but they point to the need for increased infection control and mitigation in communal spaces, including dormitories. Additionally, hospitals should ensure quality of respirators and correct training on respirator use.

**Disclosures:**

**All Authors**: No reported disclosures.

